# Visit-to-visit HbA1c variability is associated with poor prognosis in peritoneal dialysis patients with type 2 diabetes mellitus

**DOI:** 10.1186/s12882-023-03348-2

**Published:** 2023-09-29

**Authors:** Fengping Zhang, Taotao Shi, Xiaoran Feng, Yunying Shi, Guilin Zhang, Yu Liu, Ping Fu

**Affiliations:** 1 Department of Nephrology, Kidney Research Institute, West China Hospital of Sichuan University, Chengdu, China; 2 Department of Nephrology, Jiujiang NO.1 People’s Hospital, Jiujiang, China; 3 Department of Nephrology, The NO.1 Affiliatedffiliated Hospital of Nanchang University, Nanchang, China; 4 Department of Nephrology, Pingxiang People’s Hospital, Pingxiang, China

**Keywords:** HbA_1c_, Variability, Diabetes, Peritoneal dialysis, Prognosis

## Abstract

**Background:**

The prognosis of diabetic peritoneal dialysis patients is poor. HbA_1c_ serves as a crucial indicator for monitoring blood glucose control in patients with diabetes. Nevertheless, the relationship between visit-to-visit HbA_1c_ variability and prognosis in peritoneal dialysis with diabetes remains unclear.

**Methods:**

All participants were categorized into 3 groups based on the HbA_1c_ variability score (HVS), which is the frequency of 0.5% (5.5 mmol/mol) alter in visit-to-visit HbA_1c_ values. Then, the hazard ratio to HVS with all-cause mortality was analyzed using the Cox hazard model, followed by the Fine-Gray competing risk model for major adverse cardiovascular events. Subgroup and sensitivity analysis were conducted to ascertain the robustness of the findings.

**Results:**

Eight hundred twenty patients with type 2 diabetes were finally enrolled in this study from 2,855 participants with a mean age of 56.9 ± 14.6 years and a median follow-up time of 44 months [IQR: 27–70], death occurred in 496 (60.2%) individuals. Compared with the lowest category (HVS < 1/3) after being adjusted by potential confounding factors, the hazard ratio for all-cause mortality was 4.59 (3.74–5.64) and the sub-distribution hazard ratio for major adverse cardiovascular events was 1.91 (1.46–2.51) of the highest category (HVS ≥ 2/3). Subgroup interaction and sensitivity analysis, including the adjustment for variables such as time-weighted average HbA_1c_, HbA_1c_ measurement times and expansion, confirmed the reliability of the results.

**Conclusion:**

The HVS is related to the risk of poor prognosis in peritoneal dialysis with type 2 diabetes mellitus, independently of clinical multiple variables, and is a novel indicator with clinical guidance.

**Supplementary Information:**

The online version contains supplementary material available at 10.1186/s12882-023-03348-2.

## Introduction

Diabetes mellitus is one of the most prevalent causes of end-stage renal disease worldwide, and peritoneal dialysis is an essential form of renal replacement therapy. In China, diabetes (the vast majority are type 2) accounts for 19% to 22.9% of peritoneal dialysis [1]. The use of of glucose dialysate since the unpopularity of icodextrin dialysate and volume overload has led to the vulnerability of patients with diabetes to severe challenges such as blood glucose fluctuations, cardiovascular events and microvascular complications [2]. Despite gradual improvements in dialysis technology and care in recent years, diabetes individuals still have the highest mortality rate in the dialysis subgroup [3]. Previous studies have demonstrated that control of blood glucose and normalization of HbA_1c_ reduce the risk of cardiovascular events and microvascular complications in general diabetes characters [4], and have similar benefits in dialysis population [5]. However, fasting glucose and HbA_1c_ are somewhat inaccurate in dialysis due to uremic toxin, continuous exposure to glucose peritoneal dialysate and anemia [6, 7]. HbA_1c_ variability is a practical indicator for diabetes management and connected with ill consequences [8]. Interestingly, a study has identified that HbA1c variability is also related to the progression of chronic kidney diseases (CKD) [9], but the role in peritoneal dialysis is unclear. Standard deviation (SD) and coefficient of variation (CV) are commonly used to indicate HbA_1c_ variability [10]. However, in recent years, the HbA_1c_ variability score (HVS), which calculates the frequency of HbA_1c_ rise or fall by 0.5% (5.5 mmol/mol) by visit-to-visit information, has been reported to be associated with cardiovascular disease (CVD) and microvascular complications in diabetes mellitus and is more readable in clinical practice than purely statistical descriptions such as SD and CV [11]. Therefore, we sought to ascertain the correlation between the novel index and overall mortality and major adverse cardiovascular events (MACE) in peritoneal dialysis patients with diabetes by using a multicenter, large-sample database.

## Research design and methods

### Study design and participants

The study was an observational retrospective cohort study to investigate the relationship between HVS (A new indicator of HbA1c variability) and prognosis in peritoneal dialysis patients with type 2 diabetes mellitus. ALL participants were selected from a multi-center peritoneal dialysis data alliance, which is a multi-designed and continuously updated database designed to investigate various clinical dreadful events, prognosis and other risk information of Chinese peritoneal dialysis individuals. Data are recorded by each sub-center based on uniformly defined criteria and managed by dedicated personnel. The criteria were met for enrollment in our study: (1) Over 18 years old; (2) the dialysate was glucose dialysate; (3) diagnosis of type 2 diabetes at the time of starting peritoneal dialysis; (4) the first HbA_1c_ measurement was performed at 4 weeks of stable peritoneal dialysis (baseline period), otherwise there were at least 3 subsequent HbA_1c_ retests data; (5) exclusion of missing data on regression and loss of follow-up. This protocol was reviewed and authorized by the ethics committee of the local institution. The written informed consent was approved to be waived, as participant information was anonymous and did not involve any privacy.

### The baseline parameters and follow-up

The baseline period was defined as about 4 weeks of stable peritoneal dialysis. Basic parameters included: age, sex, smoking history, diabetes history, CVD history, mean arterial pressure, body mass index, etc. Laboratory data: fasting blood glucose, hemoglobin, serum albumin, alanine aminotransferase, high-density lipoprotein low-density lipoprotein, intact parathyroid hormone, C-reactive protein (CRP), residual renal function; dialysis-related data: normalized protein nitrogen appearance (nPNA), urea clearance index (Kt/*V*_urea_), dialysate to plasma creatinine concentration ratio (D/P_cr_) and so on. The study defined the follow-up time in terms of the first outcome event from the start of peritoneal dialysis to its occurrence, with an endpoint of February 2023 in event-free cases.

### Evaluation of visit-to-visit HbA1c variability

To minimize the role of the HbA_1c_ variability parameter concerning measurement frequency and to better conform to clinical practice, we adopted HVS to assess the visit-to-visit HbA_1c_ variability. HVS is defined as the proportion of the total number of individual measurements in which the HbA_1c_ has changed by 0.5% (5.5 mmol/mol) compared with the previous measurement [11]. The first HbA_1c_ reference value was the data obtained during the baseline period. To avoid the influence of multiple HbA_1c_ measurements in a short period, we stipulate that the HbA_1c_ obtained repeatedly within 3 months can only be averaged once. HVS was divided into three categories (cut value: 1/3 and 2/3) to compare the relationship with prognosis. Furthermore, we also calculated the time-weighted average HbA_1c_ for subgroup analysis by area under the of HbA_1c_ curve/time.

### Outcomes

We reviewed two endpoints of clinical interest: all-cause death and MACE. MACE was defined to include myocardial infarction, unstable angina pectoris, stroke, heart failure, vascular intervention events and other fatal cardiovascular outcomes. MACE was used for research at the time of its first appearance.

### Statistical analyses

The data were analyzed by SPSS28.0 (IBM Corporation, NY, USA) and RStudio for Windows (R version 4.0.2). Categorical variables were expressed as frequencies and percentages. The continuous variables of normal distribution were described by means and standard deviation, while the skewed distribution was described by the median quartile range (IQR). Analysis of variance, Kruskal–Wallis H test or chi-square test were selected to examine the differences between various categories. The overall survival rate was calculated by the Kaplan–Meier survival curve and Log-rank test. The Cox regression model was applied to analyze the relative hazard ratio (HR) of HVS to all-cause death. The sub-distribution hazard ratio (sHR) between HVS and MACE was performed using Fine-Gray competing risk model, and non-CVD deaths before MACE were considered as competitive risk events. Subgroup interaction tests and sensitivity analysis were used to verify the robustness of the conclusions. The *p*-value for linear trend across multiple groups was expressed as the *p* for tend, and the *p*-value less than 0.05 was regarded as statistically significant.

## Results

### Baseline characteristics

**Fig. 1:**
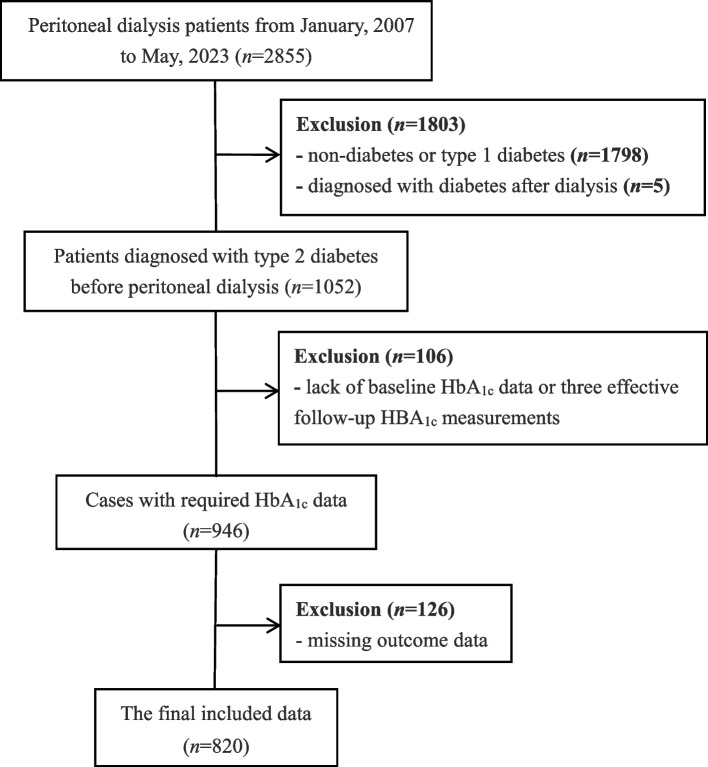
Flow-chart of the participant’s election

As shown in Fig. 1, 820 patients with type 2 diabetes were finally enrolled in this study from 2,855 participants in four peritoneal dialysis centers in China. The mean age at baseline was 56.9 ± 14.6 (range: 18–85), and 424 (51.7%) were male, the average HbA_1c_ at baseline was 7.0 ± 2.3% (53.0 ± 24.6 mmol/mol). The median number of measurements was 5.5 (IQR: 4.6, 7.0), and the mean HbA_1c_ follow-up was 7.4 ± 2.5% (57.7 ± 26.8 mmol/mol). For the details of HbA_1c_ as shown in Supplementary data. According to the classification of HVS values, 428 (52.2%) were found with HVS < 1/3, 254 (31.0%) with 1/3 ≤ HVS < 2/3, and 138 (16.8%) with HVS ≥ 2/3. The baseline characteristics of different HVS categories as shown in Table 1. The results indicated that the increase in HVS was associated with older age, larger body mass index, higher baseline HbA_1c_ levels, increased hemoglobin levels and more baseline CRP levels. It is worth pointing out that the duration of diabetes history, the difference in the frequency of HbA_1c_ measurement and the proportion of insulin therapy was not statistically significant.

**Table 1:** Baseline characteristics of different categories HVS in peritoneal dialysis patients with diabetes

	**All patients** **(*n* = 820)**	**HVS < 1/3** **(*n* = 428)**	**1/3 ≤ HVS < 2/3** **(*n* = 254)**	**HVS ≥ 2/3** **(*n* = 138)**	***p* for tend**
Age (years)	56.9 ± 14.6	55.8 ± 14.9	58.2 ± 14.2	60.1 ± 13.7	< 0.001
Sex, Male, *n* (%)	424 (51.7)	224 (52.3)	130 (51.2)	70 (50.2)	0.706
Smoking, *n* (%)	204 (24.6)	102 (23.8)	64 (25.2)	38 (27.5)	0.381
Drinking, *n* (%)	112 (13.7)	54 (12.6)	36 (14.2)	22 (15.9)	0.328
Cardiovascular disease history, *n* (%)	266 (32.4)	136 (31.8)	90 (35.4)	40 (28.9)	0.848
Hypertension, *n* (%)	670 (81.7)	354 (82.7)	202 (79.5)	114 (82.6)	0.714
Drugs					
Insulin, *n* (%)	686 (83.7)	362 (84.6)	212 (83.5)	112 (81.2)	0.353
Statins, *n* (%)	178 (21.7)	98 (22.9)	52 (20.5)	28 (20.3)	0.427
Antiplatelet, *n* (%)	250 (30.5)	112 (29.0)	92 (36.2)	34 (24.6)	0.527
Hypertensive agent, *n* (%)	666 (81.2)	340 (79.4)	214 (84.3)	112 (81.2)	0.370
Mean arterial pressure (mmHg)	97.2 ± 12.1	96.6 ± 11.8	97.3 ± 12.0	97.8 ± 12.9	0.547
Body mass index (kg/m^2^)	21.9 ± 3.5	21.6 ± 3.3	22.2 ± 3.6	22.8 ± 4.1	0.010
Mean time of diabetes history (years)	15.5 ± 6.7	15.2 ± 5.1	15.6 ± 5.8	16.0 ± 7.1	0.153
Frequencies of HbA_1c_ measurement (per patient)	5.5 (4.6, 7.0)	5.6 (4.3, 7.2)	5.3 (4.2, 7.1)	5.2 (4.0, 6.4)	0.109
HbA_1c_ at baseline (%)	7.0 ± 2.3	6.8 ± 2.1	7.4 ± 2.8	8.0 ± 3.2	< 0.001
HbA_1c_ at baseline (mmol/mol)	53.0 ± 24.6	51.1 ± 23.0	57.7 ± 30.2	64.3 ± 35.1	< 0.001
Fasting blood-glucose (mmol/L)	8.1 ± 2.7	7.8 ± 2.5	8.0 ± 2.8	8.2 ± 3.1	0.146
Hemoglobin (g/L)	90.6 ± 10.4	88.8 ± 10.1	90.1 ± 10.5	92.3 ± 9.8	< 0.001
Alanine aminotransferase (U/L)	23.2 ± 8.0	22.9 ± 8.1	23.5 ± 7.8	24.1 ± 8.6	0.072
Serum albumin (g/L)	35.0 ± 5.3	34.9 ± 5.1	35.0 ± 5.4	34.8 ± 5.2	0.807
High-density lipoprotein (mmol/L)	1.4 ± 0.4	1.4 ± 0.4	1.4 ± 0.5	1.4 ± 0.5	0.143
Low-density lipoprotein (mmol/L)	3.1 ± 1.2	3.0 ± 0.9	3.1 ± 1.1	3.2 ± 1.4	0.072
Intact parathyroid hormone (pg/ml)	250 ± 110	254 ± 109	246 ± 127	259 ± 104	0.953
Residual renal function (ml/min)	3.8 (1.2, 9.2)	3.7 (1.1, 7.1)	3.8 (1.6, 9.9)	3.9 (1.8, 10.4)	0.095
C-reactive protein (mg/L)	5.9 (4.3, 8.0)	5.8 (4.2, 7.5)	6.1 (4.6, 7.8)	6.4 (4.5, 8.3)	< 0.001
nPNA (g/kg/day)	1.1 ± 0.2	1.1 ± 0.3	1.0 ± 0.2	1.1 ± 0.2	0.460
D/P_cr_	0.7 ± 0.2	0.7 ± 0.3	0.7 ± 0.1	0.7 ± 0.2	0.389
Total Kt/*V*_urea_	1.8 ± 0.5	1.8 ± 0.5	1.8 ± 0.4	1.8 ± 0.4	0.246

*Abbreviation*: *nPNA* normalized protein nitrogen appearance, *D/P*_cr_ dialysate to plasma creatinine concentration ratio, Kt/*V*_urea_ urea clearance index

### HVS and outcome

**Fig. 2:**
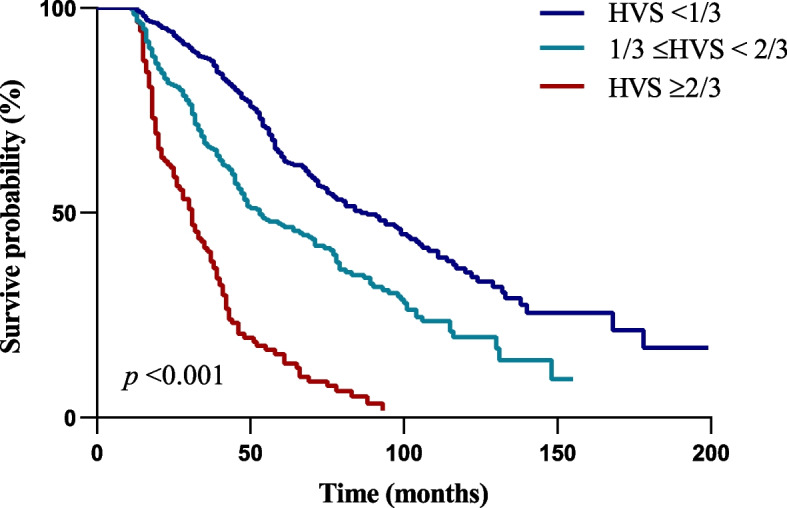
Survive curve of all-cause mortality in different HVS categories

**Fig. 3:**
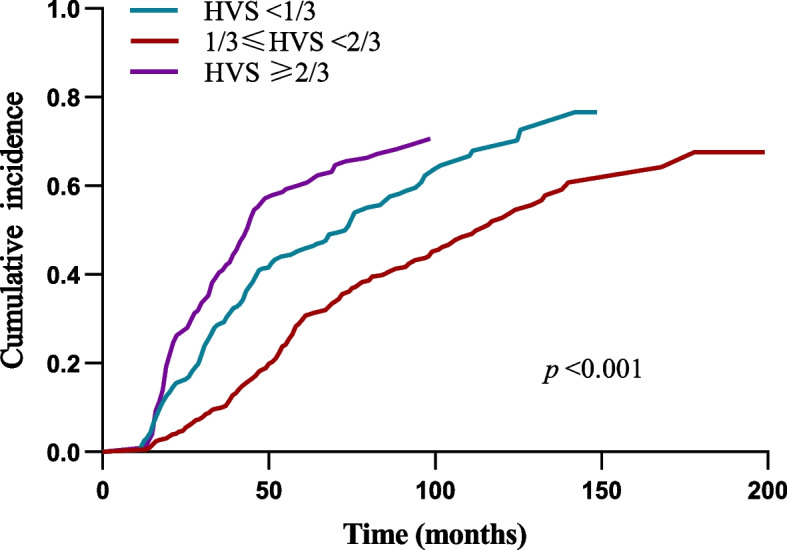
Association between HVS and MACE (Fine-Gray competing risk model)

The median duration was 44 (12–198) [IQR: 27, 70] months, when all participants were followed up to the end. Deaths from all causes occurred in 494 (60.2%) patients, including 340 (41.5%) deaths from CVD disease, accounting for 68.8% of all-cause deaths. MACE occurred in 390 (47.6%) of all participants. The Kaplan–Meier survival curve by Log-rank test showed that the overall survival rate decreased significantly with the rise of HVS (*p* < 0.001) (Fig. 2). Fine-Gray competing risk model test displayed that the MACE cumulative incidence of HVS ≥ 2/3 category was significantly higher than that of the other two categories (*p* < 0.001) (Fig. 3).

For further analysis, set the lowest category (HVS < 1/3) as the reference, unadjusted Cox regression analysis suggested that the HR of all-cause mortality and the sHR of MACE with HVS ≥ 2/3 category were 4.87 [95% confidence intervals (CIs): 4.02–5.89, *p* < 0.001] and 2.13 (95% CI:1.67–2.71, *p* < 0.001), respectively. Adjusted for a multivariate model, the HR was 4.59 (95% CI:3.74–5.64, *p* < 0.001), and the sHR was 1.91 (95% CI:1.46–2.51, *p* < 0.001), (Tables 2 and 3). These findings implied that the increase in HVS was distinctly related to the inferior outcomes of peritoneal dialysis individuals.

**Table 2:** Cox regression analysis of HVS and all-cause mortality

	Unadjusted Model		Model 1		Model 2	
	HR (95%CI)	*p*-value	HR (95%CI)	*p*-value	HR (95%CI)	*p*-value
HVS < 1/3	1.0 (reference)		1.0 (reference)		1.0 (reference)	
1/3 ≤ HVS < 2/3	1.73 (1.46—2.04)	< 0.001	1.59 (1.33—1.91)	< 0.001	1.62 (1.35—1.94)	0.002
HVS ≥ 2/3	4.87 (4.02—5.89)	< 0.001	4.48 (3.66—5.49)	< 0.001	4.59 (3.74—5.64)	< 0.001
*p* for trend	< 0.001		< 0.001		< 0.001	

Model 1: adjusted for time-weighted average HbA_1c_

Model 2: adjusted for time-weighted average HbA_1c_ and other factors (age, sex, cardiovascular disease history, body mass index, hemoglobin, albumin and C-reactive protein)

**Table 3:** Association between HVS and MACE (Fine-Gray competing risk model)

	Unadjusted Model		Model 1		Model 2	
	sHR (95%CI)	*p*-value	sHR (95%CI)	*p*-value	sHR (95%CI)	*p*-value
HVS < 1/3	1.0 (reference)		1.0 (reference)		1.0 (reference)	
1/3 ≤ HVS < 2/3	1.58 (1.29—1.94)	< 0.001	1.51 (1.22—1.86)	< 0.001	1.44 (1.14—1.81)	0.002
HVS ≥ 2/3	2.13 (1.67—2.71)	< 0.001	2.04 (1.58—2.62)	< 0.001	1.91 (1.46—2.51)	< 0.001

Model 1: adjusted for time-weighted average HbA_1c_

Model 2: adjusted for time-weighted average HbA_1c_ and other factors (age, sex, cardiovascular disease history, body mass index, hemoglobin, albumin and C-reactive protein)

### Subgroup and sensitivity analysis

**Fig. 4:**
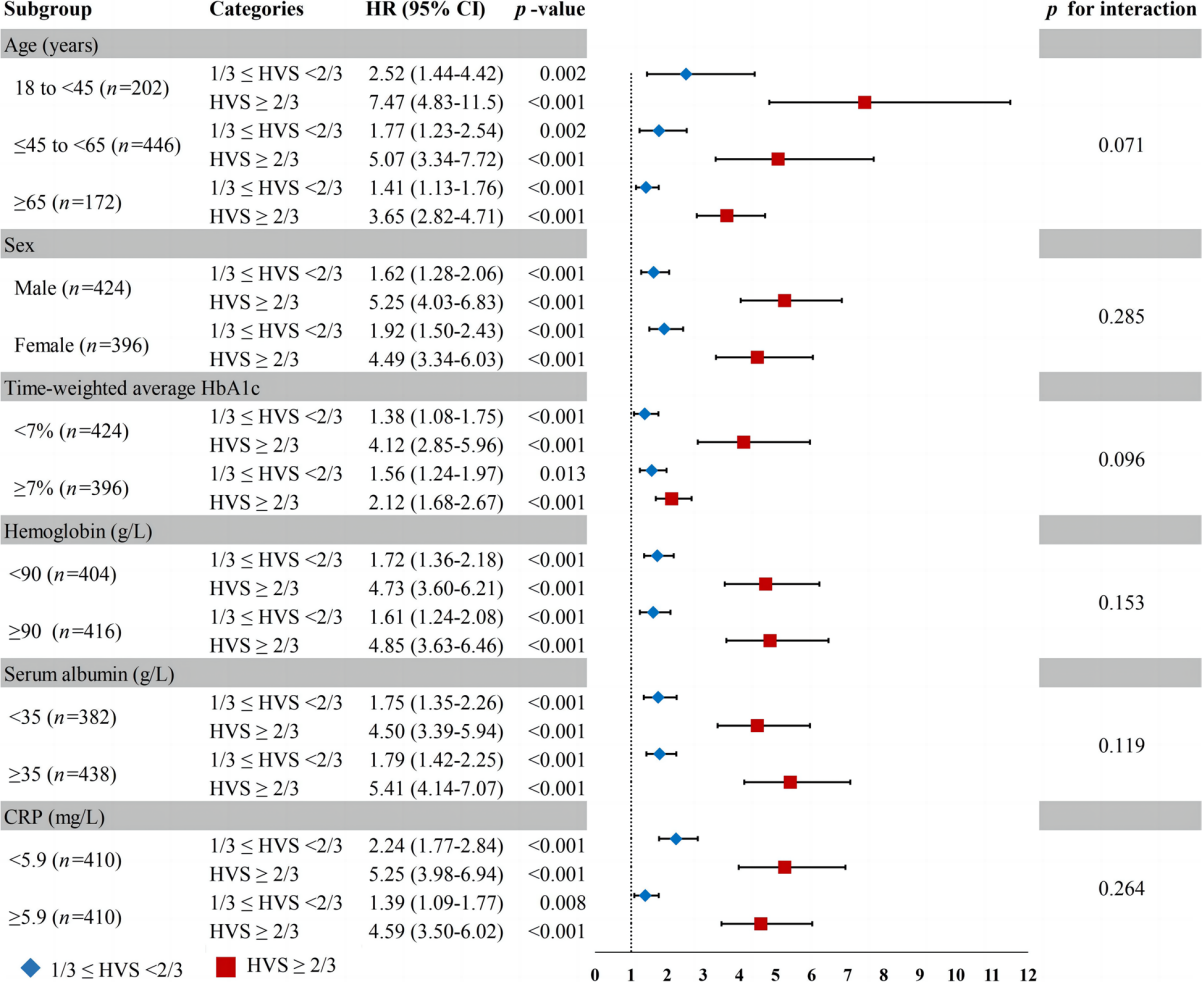
Forest plot for subgroup analysis of HVS and all-cause mortality (refer to HVS < 1/3)

**Fig. 5:**
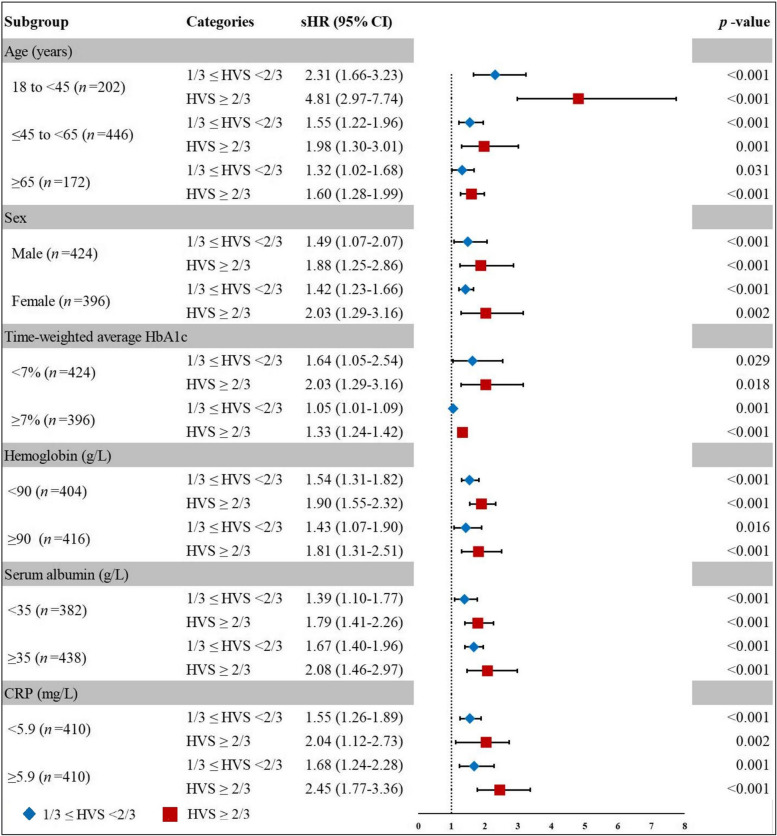
Forest plot for subgroup analysis of HVS and MACE (refer to HVS < 1/3)

For the sake of verifying the robustness of the correlation between HVS and outcomes, we performed a subgroup analysis. After multivariate adjusted, the results showed that for the age subgroup, the risk association between HVS and all-cause death was enhanced in patients less than 45 years old, while it was weakened in the subgroup based on time-weighted average HbA_1c_ ≥ 7.0% (53 mmol/mol). However, the overall trend remained largely consistent. When stratified analyses were performed for gender, baseline hemoglobin, baseline albumin and baseline CRP, the interaction test showed that all the *p*-value for interaction of each subgroup were over 0.05, indicating that the results were not significantly dependent on these variables (Figs. 4 and 5).

### Time-dependent ROC curve

**Fig. 6:**
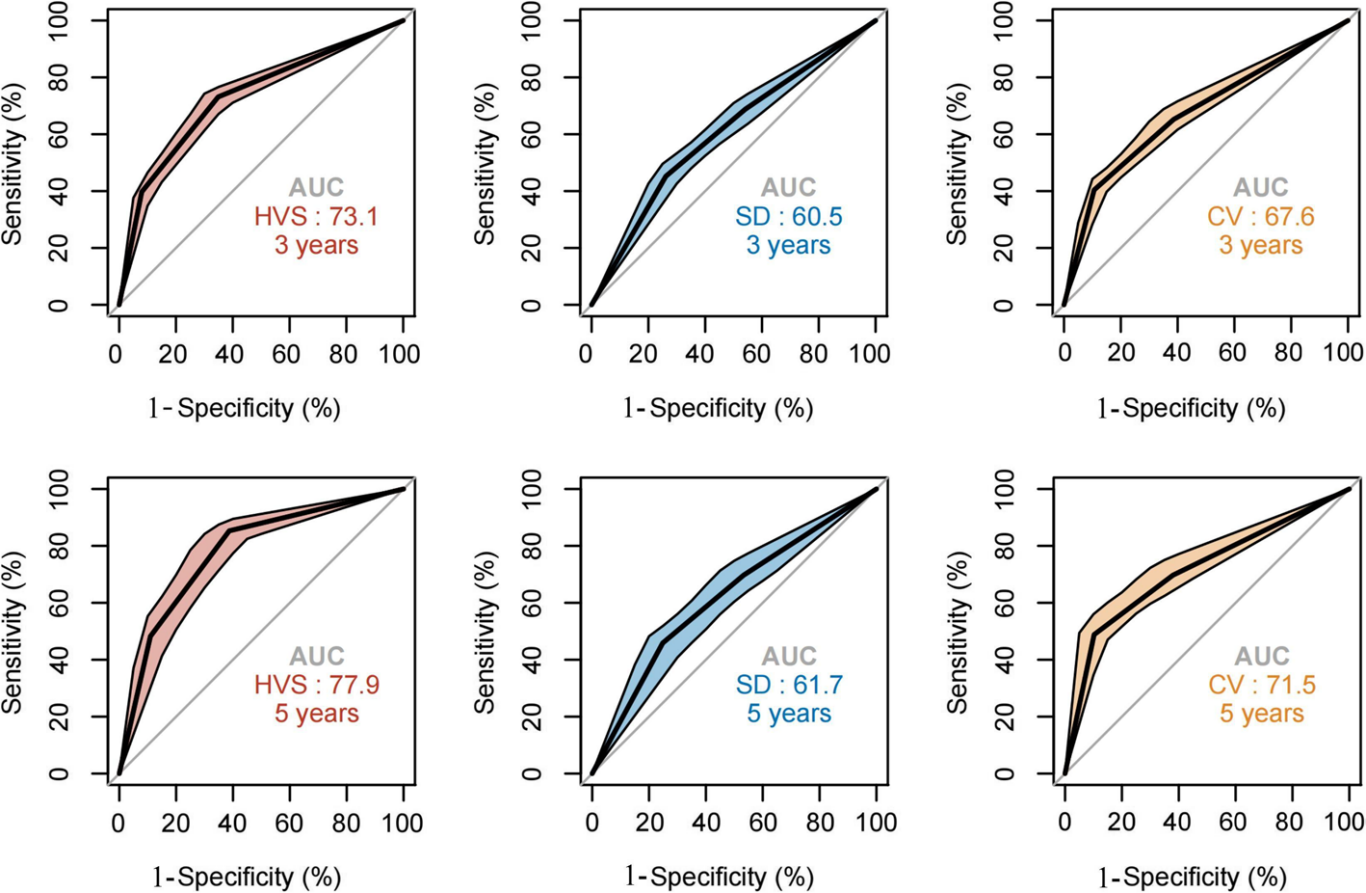
Time-dependent ROC curve of HVS, SD and CV of HbA_1c_ with all-cause mortality

By analyzing time-dependent receiver operator characteristic curves, we examined the ability of HVS, SD and CV to predict all-cause mortality. As shown in Fig. 6, regardless of the predicted value of 3 years or 5 years, the performance of HVS is superior to that of SD and CV.

## Discussion

We analyzed the relationship between visit-to-visit HbA_1c_ Variability and adverse endpoint events in peritoneal dialysis with type 2 diabetes and used HVS as a new index to evaluate HbA_1c_ variability for the first time. It is proved that frequent fluctuations of HbA_1c_ adjacent to measurements are independent risk factors for all-cause mortality and MACE in patients with type 2 diabetes. Our study showed that after adjustment for several parameters, including time-weighted average HbA_1c_, patients with HVS ≥ 2/3 had a substantially increased risk of detrimental outcomes. The HR of all-cause mortality and sHR of MACE were 4.59 and 1.91, respectively, compared with patients with HVS < 1/3. Additionally, we adjusted several unique confounding factors of peritoneal dialysis, such as residual renal function, normalized protein nitrogen appearance, and dialysis adequacy. Rich subgroup interaction effects confirmed that there was no remarkable alteration in the trend of high HVS classification associated with a high risk of harmful endpoint events among diverse individuals, though the sample size might not have been sufficient to achieve the desired *p*-value in the stratified analyses. Simultaneously, the efficiency in predicting outcomes of HVS was superior to SD and CV.

As is known to all, glucose level, hemoglobin and the interaction time between them will affect the variation of HbA_1c_ [12], and continuous exposure to glucose dialysate, low levels of albumin and hemoglobin and other states are inevitable in peritoneal dialysis, so we focused on correcting the influence of these factors in this study. It is worth noting that for subgroups with a time-weighted average HbA_1c_ ≥ 7.0% (53 mmol/mol), although a positive association was observed between HVS and detrimental foreground, the correlation seemed to be diluted. We deem that higher HbA_1c_ levels often indicate more peritoneal glucose exposure and peritoneal injury, resulting in high peritoneal transport, reduced ultrafiltration capacity, aggravating volume load and contributing to higher mortality, which diminish the relationship between HVS and prognosis to a certain extent. Interestingly, we found an enhanced association between HVS and the risk of death in peritoneal dialysis patients under 45 years of age. As previously reported, young patients with diabetes have fewer regular visits, and a large proportion of these population are in the HbA_1c_ measurement mode with discrete (SD at measurement intervals in the high quartile), which may miss an appropriate opportunity for readmission [13]. Recently accumulated evidence suggests that compared with the characteristics of the geriatric, such as large blood glucose fluctuations, the occurrence of abnormal blood glucose in young patients may be more indicative of the decline of organ function or unhealthy lifestyle, which leads to a higher mortality risk [14]. In brief, our study indicates that stable HbA_1c_ control still has a substantial benefit in peritoneal dialysis patients as well.

In the general population, HbA_1c_ variability is undoubtedly associated with MACE, microvascular disease and all-cause mortality. Oxidative stress, endothelial cell dysfunction, hypoglycemia events and cumulative epigenetic modification caused by blood glucose fluctuations are considered to be the potential causes of this relevance [15]. In patients with chronic kidney disease and dialysis, there are few studies on blood glucose control and prognosis. Data on hemodialysis from Japan suggests that a high ratio of Glycated Albumin to Glycated Hemoglobin is correlated with a higher mortality rate [16]. Another study of 2,798 peritoneal dialysis cases found that patients with HbA_1c_ ≥ 8.0% (63 mmol/mol) had higher all-cause mortality, but the study also noted that HbA_1c_ was vulnerable to albumin, inflammation, and hemoglobin [5]. About the variability of HbA_1c_ and the prognosis of dialysis patients, *Afghahi H, *et al. [17] demonstrated that the inflation risk of all-cause mortality in peritoneal dialysis patients with diabetes was prominently connected with the increase of CV in HbA_1c_, which is consistent with our conclusion, but 48.9% of the patients included in their study were measured only twice, and it would weaken the accuracy of CV.

Our finding possesses certain advantages. Firstly, We have effectively integrated large-scale multicenter data, long follow-up time and clinically important negative outcomes were recorded, and confirmed by a variety of stratified and robust analyses. And we demonstrated consistent outcomes about two commonly occurring adverse events. Secondly, all subjects were tracked by their first HbA_1c_ measurements from balanced dialysis, so the dialysate glucose exposure was stable and there was no loss of disease progression after dialysis. In addition, the study stems from real-world data of Chinese peritoneal dialysis patients, so the results can be directly translated into clinical practice. Finally, the HVS, rather than SD or CV, is more likely to represent a trend of statistical discretization with no clinical guidance value, but it is more clinically readable when adopting HVS. Nephrologists can examine individual HbA_1c_ fluctuation curves and determine that patients with frequent differences of more than 0.5% (5.5 mmol/mol) in pre- and post-measurement results are at high risk. To put it simply, our study demonstrates from a practical point of view that frequent fluctuations in HbA_1c_ are a hazard factor for worse outcomes. Meanwhile, these findings indicate that HVS can be used as a potential clinical parameter to guide the management of diabetic peritoneal dialysis patients.

This study still has some limitations. First of all, as an observational retrospective cohort study, it is possible that some potential confounders (such as irregular measurement, hypoglycemic events or nursing quality) may limit the use of HVS, and we cannot point out the causal relationship between variability itself and prognosis. Secondly, due to the shortcomings of follow-up data, we failed to fully evaluate the role of changes in peritoneal dialysis prescription and accurate hypoglycemic drug dosage in the correlation between HVS and outcomes. In addition, participants with diabetes in this study were not specifically analyzed according to HbA_1c_, socio-demographic or non-diabetes medical-related segmentation, which may have broad applications for evaluating clinical prognosis and deserve further investigation [18]. Finally, on account of the limitation of the numbers included in HbA_1c_, almost every participant in the study was followed up for more than one year, early deaths and some incomplete data were not taken into account accordingly. To some extent, this limits the value of early mortality forecast, although we know that the death of dialysis patients within one year may be susceptible to age, cardiac function, blood pressure, volume load and other basic states [19]. However, this study is of great significance for the judgment of medium- and long-term outcomes.

## Conclusion

In conclusion, our research indicates that a higher visit-to-visit HVS in peritoneal dialysis patients with diabetes is independently related to an increased risk of all-cause mortality and MACE.

### Supplementary Information

**Additional file 1: Supplementary data Fig. S1.** The HbA1c  at the baseline and follow-up.

## Data Availability

The data that supports these results of our research are available from the corresponding author on acceptable demand.
