# Supplementary material for: Visit-to-visit HbA1c variability is associated with poor prognosis in peritoneal dialysis patients with type 2 diabetes mellitus
Source: BMC Nephrol. 2023 Sep 29;24:288. doi: 10.1186/s12882-023-03348-2 (PMC10542698; doi:10.1186/s12882-023-03348-2)

SUPPLEMENTARY DATA

Fig.S1 The HbA1c at the baseline and follow-up

1. The mean and standard deviation of HbA1c and frequency distribution of each segment at baseline; B. The follow-up HbA1c values classified by the number of measurements


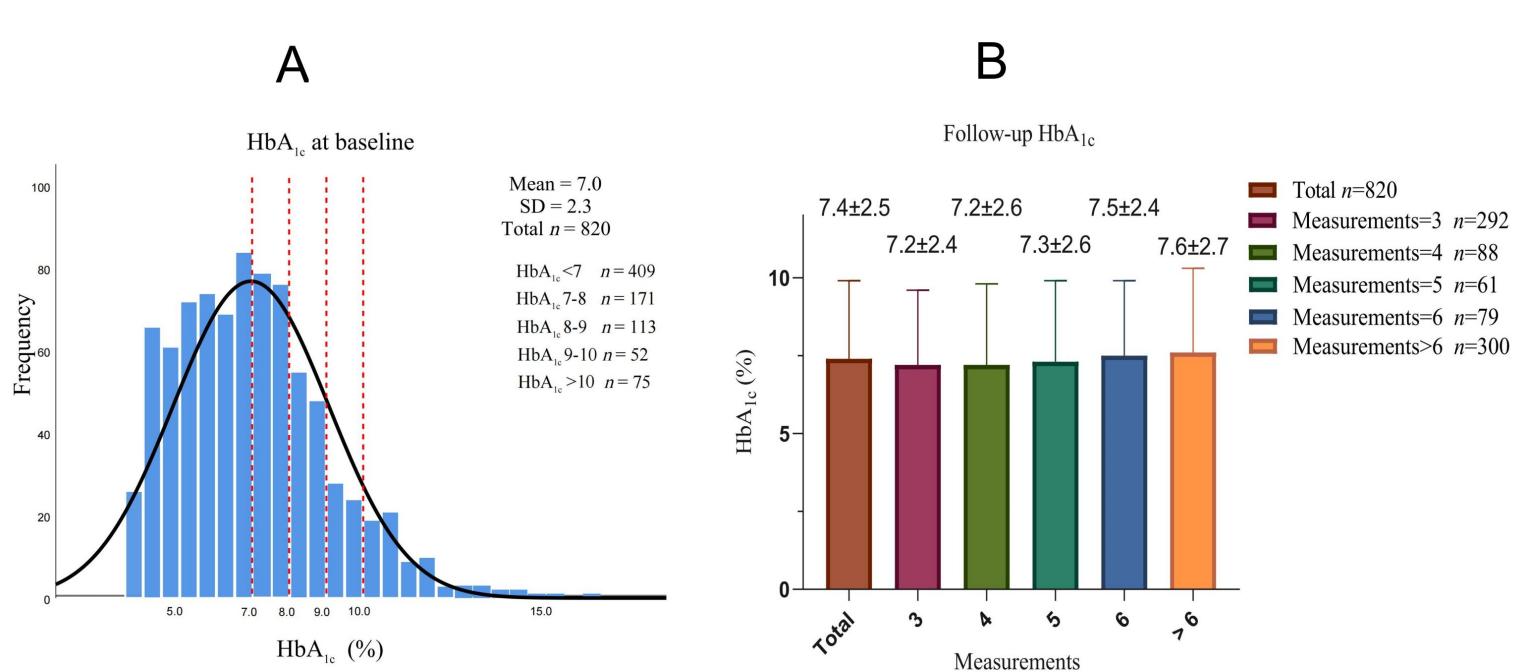

Supplement: Supplementary file 1 — Additional file 1: Supplementary data Fig. S1. The HbA1c at the baseline and follow-up. [file 12882_2023_3348_MOESM1_ESM.doc]
